# Homobifunctional imidoester-modified zinc nano-spindle attenuated hyphae growth of *Aspergillus* against hypersensitivity responses

**DOI:** 10.1016/j.isci.2022.105922

**Published:** 2023-01-11

**Authors:** Huifang Liu, KeLun Zhang, Yoon Ok Jang, Zhen Qiao, Jie Jin, Thuy Nguyen Thi Dao, Bonhan Koo, Chang Ook Park, Yong Shin

**Affiliations:** 1 Department of Biotechnology, College of Life Science and Biotechnology, Yonsei University, Seoul 03722, Republic of Korea; 2 Department of Dermatology, Severance Hospital, Cutaneous Biology Research Institute, Yonsei University College of Medicine, Seoul, Korea; 3 Brain Korea 21 PLUS Project for Medical Science, Yonsei University College of Medicine, Seoul, Korea; 4 Department of Chemical and Biomolecular Engineering, Yonsei University, Seoul 03722, Republic of Korea

**Keywords:** Pathogenic organism, Treatment, Nanomaterials

## Abstract

Fungi cause various forms of invasive fungal disease (IFD), and fungal sensitization can contribute to the development of asthma, asthma severity, and other hypersensitivity diseases, such as atopic dermatitis (AD). In this study, we introduce a facile and controllable approach, using homobifunctional imidoester-modified zinc nano-spindle (HINS), for attenuating hyphae growth of fungi and reducing the hypersensitivity response complications in fungi-infected mice. To extend the study of the specificity and immune mechanisms, we used HINS-cultured *Aspergillus* extract (HI-AsE) and common agar-cultured *Aspergillus* extract (Con-AsE) as the refined mouse models. HINS composites within the safe concentration range inhibited the hyphae growth of fungi but also reduce the number of fungal pathogens. Through the evaluation of lung and skin tissues from the mice, asthma pathogenesis (lung) and the hypersensitivity response (skin) to invasive aspergillosis were least severe in HI-AsE-infected mice. Therefore, HINS composites attenuate asthma and the hypersensitivity response to invasive aspergillosis.

## Highlights

• Introduce a facile and controllable approach with HINS
• HINS composite could inhibit the growth of hyphae
• HINS composite could reduce the number of fungal pathogens
• HINS composites attenuate hypersensitivity response to invasive aspergillosis

## Graphical abstract

**Figure:**
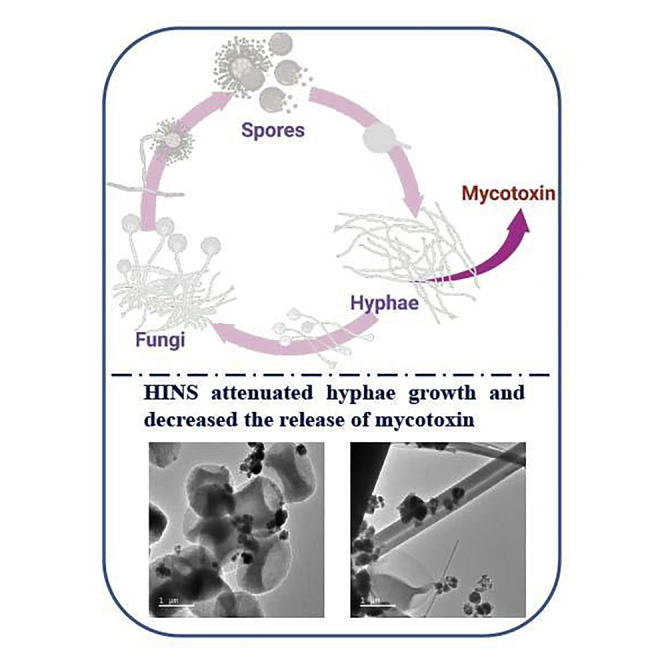

## Introduction

Fungi are widely distributed in the environment, and although the majority of fungi are generally non-pathogenic to humans,^1^^,^^2^^,^^3^ some cause various forms of the disease. In particular, superficial infestations from fungi have been diagnosed more frequently, with an increasing number of patients reported.^4^^,^^5^^,^^6^ Fungal allergy can contribute to asthma severity and other hypersensitivity diseases, such as atopic dermatitis (AD), which is closely correlated with allergic rhinitis and allergic asthma, which normally occur in children and young adults.^7^^,^^8^ *Aspergillus fumigatus* is the most common cause of invasive fungal diseases (IFDs), which constitute a leading cause of morbidity and mortality in recipients with malignant disease undergoing blood, bone marrow, stem cell transplantation, or solid organ transplantation.^9^^,^^10^^,^^11^ Poorly maintained ventilation and water systems in medical facilities and contamination of instruments are also reported as potential causes of infection.^10^

A plethora of fungal co-infections in patients affected and recovering from coronavirus disease 2019 (COVID-19) have been documented worldwide, raising concern for an outbreak of life-threatening fungal complications in patients with COVID-19.^12^^,^^13^ Oxygen delivery is crucial for intensive care unit patients infected with COVID-19. The oxygen must be both highly sterilized and purified and requires sterile water for humidification. If oxygen is administered without being humidified, it dries out the mucous membrane and damages the lungs’ inner lining.^14^^,^^15^ The phlegm becomes viscous and is difficult to expel. Inhalation of fungi spores by patients weakens the immune system and can lead to infection of the sinuses, lungs, skin, and soft tissue, thrombosis, necrosis, inflammation, and even death, highlighting the need to optimize prophylaxis against invasive fungal infections and increase treatment choices.^16^^,^^17^

Numerous new prophylaxis and therapeutic approaches have been explored to resist fungi infection. Many bionanomaterial research teams are committed to studying advanced biomedical candidates based on traditional antibiotics, and safety equipment.^18^^,^^19^^,^^20^ Cumulating *in vitro* evidence suggests that many of these biomaterial interactions result in synergistic antimicrobial effects with antibiotics (a positive side effect).^21^^,^^22^^,^^23^ The proposed general mechanisms for the ability of nanomaterials to overcome antimicrobial resistance include the following: (1) Membrane damage: nanomaterials tend to rupture the cell membrane and damage the cell structure^24^^,^^25^^,^^26^; (2) reactive oxygen species production: many nanomaterials induce the production of reactive oxygen species, which impair microbial metabolism and damage protein, membrane lipids, and DNA^26^; (3) ion dissolution: released ions, such as silver ions, could attack biomolecules (e.g., thiol-rich proteins) directly^18^^,^^27^; (4) enzyme-mimicking performance: the peroxidase-like activity of nanomaterials, such as palladium nanocrystals and iron oxide, enhances their bactericidal properties; and (5) biofilm eradication: some metal oxide nanoparticles can eradicate biofilms because of their strong redox reaction capabilities that perturb the cell metabolism.^24^^,^^25^^,^^26^ However, antifungal prophylaxis is linked to drug-resistant fungal infections and probably results in disruption of the microbiota balance. These fungi could invade the bloodstream and cause fatal invasive fungal infections (a negative side effect).^24^^,^^25^^,^^26^ Moreover, several techniques using metal oxide nanoparticles in various biomedical sciences have been studied that have a great expectation on prevention and therapy of infections^20^^,^^28^

In this study, we report an approach for attenuating human responses to invasive aspergillosis in a mouse model through building functional nanomaterials for prophylaxis. *In vitro* study of homobifunctional imidoester-modified zinc nano-spindle (HINS) material has just proved enhanced biocompatibility, antibiotic efficacy, and blood coagulation reduction in the field of antibiotics. Drug resistance development and pathogenicity are associated with biofilm formation by fungal pathogens,^11^^,^^29^^,^^30^ so we wondered whether we could develop a prophylactic measure against pathogenic fungi by promoting nanomaterial composite substances for packaging of daily stuff, construction materials, and medical instruments to attenuate hypersensitivity responses in lung and skin. We found that the 2.5% mixture ratio of HINS and culture medium would decrease the growth of spores, and the 5.0% mixture ratio of HINS effectively inhibited the hyphae growth of fungi for 90 h and even longer under subtropical conditions suitable for fungal growth. Next, to evaluate the histomorphological effects of HINS on lung and skin hypersensitivity mouse models were established by intranasal (i.n.) and topical exposure to *A. fumigatus*, called homobifunctional imidoester-modified zinc nano-spindle agar-cultured *Aspergillus* extract (HI-AsE). Through the evaluation of sensitization, dermatitis severity, and histological analysis of lung and skin tissue, we found that asthma pathogenesis (lung) and the hypersensitivity response (skin) to invasive aspergillosis were less severe in HI-AsE-infected mice compared with common agar-cultured *Aspergillus* extract (Con-AsE)-infected mice. The Con-AsE group showed significantly greater epidermal and dermal thickness than the HI-AsE group. Meanwhile, the HI-AsE group showed less inflammation compared to the Con-AsE group. In this light, we suggest that HINS composites for daily packaging stuff, construction materials, and medical instruments would help decrease pathogenic fungal infection in the lung and skin inflammation induced by i.n. and topical exposure to mold.

## Results and discussion

### Principle of HINS composite against fungal infection

**Scheme 1:**
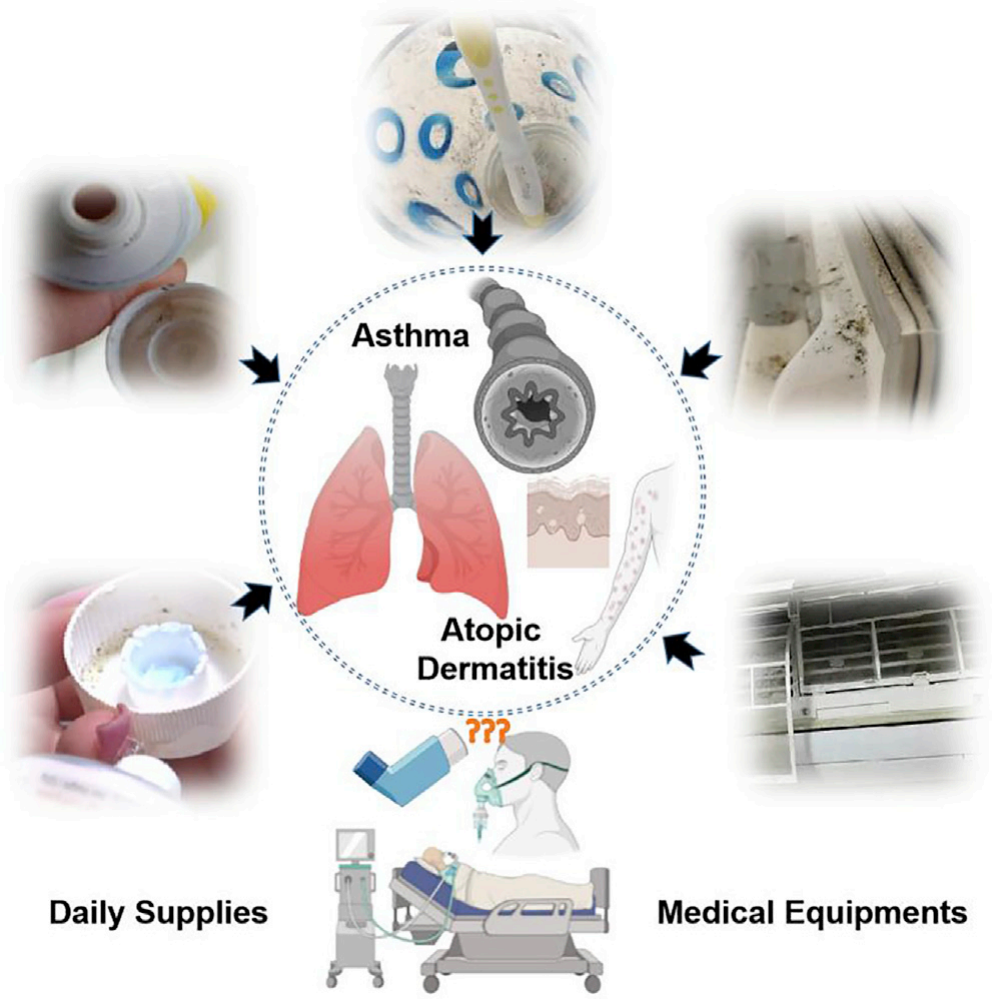
Development of prophylactic against pathogenic fungi by promoting nanomaterial composite substances for packaging of daily stuff, construction materials, and medical instrument

**Figure 1:**
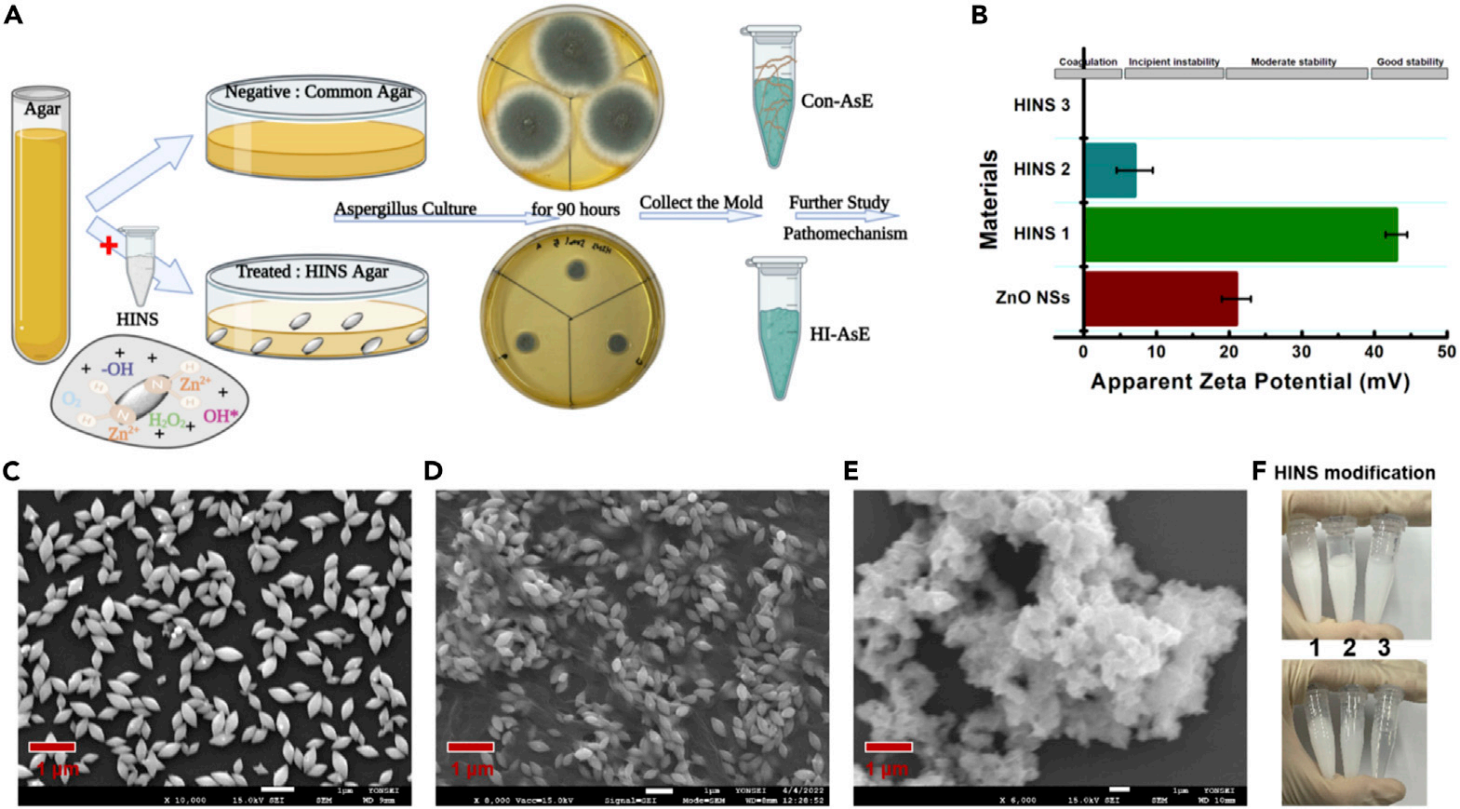
Function schematic and chemical characterizations of tested HINS (A) Flow diagram of the HINS-treated *Aspergillus* collection. (B) Zeta potential of HI-modified ZnO nanomaterials with the graphs of the soluble series (HI concentrations)-modified HINS composite that has been kept standing for 30 min. (C) SEM images of synthesized ZnO NS (approximately 200 nm) in uniform nano-spindle structure. (D) SEM images of the proper HI-modified ZnO NS (HINS). (E) SEM images of ZnO NS modified by HI at an excessively high concentration (HINS_cluster). (F) Photos of the solution status for modification control. Each zeta potential value indicates ±SE of duplicate independent experiments.

**Figure 2:**
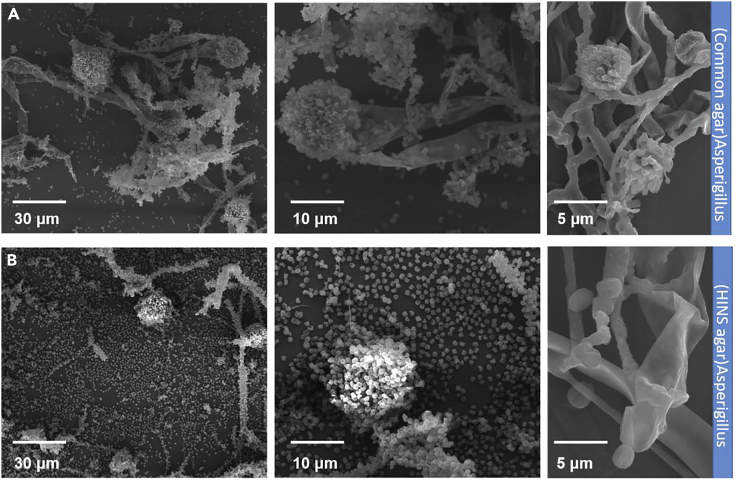
Morphology of *Aspergillus* SEM images of *Aspergillus* under different culture conditions. (A) Common agar growth: *Aspergillus* exhibits strong and numerous hyphae. (B) HINS agar growth: *Aspergillus* exhibits fragile and fewer hyphae (room temperature dry).

To examine whether HINS composite administered i.n. and i.p. attenuates fungal growth and activity (Scheme 1), we established the culture medium composition for HINS and collected the cultured *Aspergillus*, Con-AsE, and HI-AsE, respectively (Figure 1A and Table 1). HINS was prepared through the facile modified hydrothermal method (Table 1). Owing to the stability and biocompatibility of HINS being the main factors of the enhanced efficiency, we used different concentrations (1–10 mg/mL) of HI (DMS) to modify the ZnO NSs for 20 min. To substantiate the analysis of the concentration-effect reaction, the zeta potentials of the HINS were measured (Figure 1B). The apparent zeta potential of ZnO NSs reached +21 mV, which revealed the incipient instability of our fundamental materials. Moreover, the apparent zeta potential of 1 mg/mL of the modified HINS composite changed to +44 mV, which indicated that HINS-1 gained electrokinetic potential and had good stability. However, as the concentration of HI increased, the solution became sticky, and the apparent zeta potential of HINS-2 was lower than that of ZnO NSs. Moreover, HINS-3 exhibited no significant zeta potential. These changes demonstrated that the energy shift after proper HI modification would provide the composite with more energy and activity (Figure 1B). Meanwhile, we observed the morphology of the materials using an SEM instrument: The uniform particles of ZnO NSs and the different concentrations of modified HINS could be shown clearly in the SEM images. Compared with the smooth and clean shape of ZnO NSs (Figure 1C), the HINS seems to be wearing a bridal gown and has a soft surface (Figure 1D). The susceptibility of HI to hydrolysis indicated that the HI modification could help to protect the modified materials in an independent stable condition. However, when the concentration of HI was too high, the ZnO NSs mingled and aggregated, and their morphology was obscured (Figure 1E). Thereby, the optimal concentration of modified HI on ZnO NSs is quite important for preparing its conjugation. Meanwhile, the photos (Figure 1F) recorded the reaction, showing that a high concentration of HI would overreact with the ZnO NSs; 10 mg/mL of HI would dissolve/release materials out of the solid phase. All these indicated that the relative electron beam density caused the surface modification of HI of ZnO NSs, and the proper surface energy could lead to enhanced applications of materials. Next, the fungi growing in the HINS-treated agar showed a different morphological tendency (Figure 2). We noticed that *Aspergillus* grown on the common agar exhibited strong hyphae (Figure 2A) compared with its fragile hyphae on HINS agar (Figure 2B). Based on the strong hyphae growth and plump spores of *Aspergillus*, the HINS could attenuate the hyphae growth and lyse the spores of *Aspergillus*.

### HINS attenuates the hyphae growth of fungi

**Figure 3:**
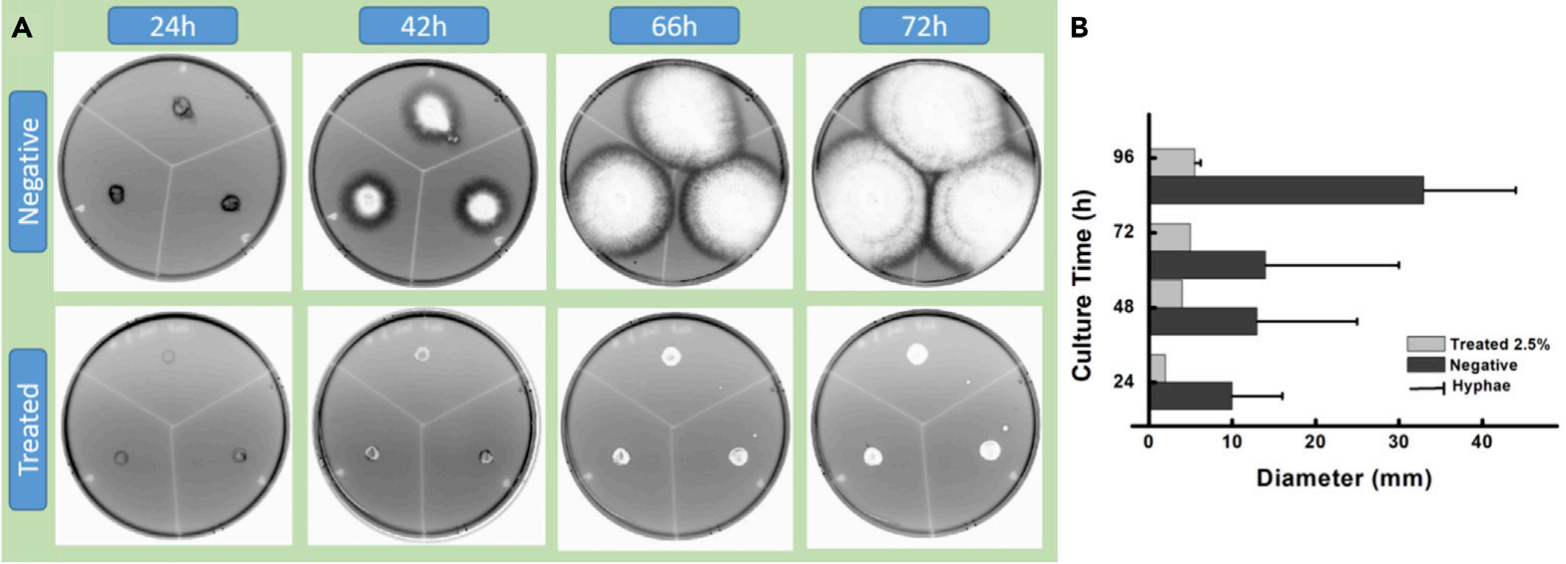
HINS inhibits the fungi growth (A) Photos of *Aspergillus* colony in HINS-treated agar. (B) Proportion summary of the growth rate of *Aspergillus* colony treated with HINS and negative control through the spread plate method for studying the antifungal efficiency of HINS (mg/mL, mixed with agar medium, the bright parts are the spore and the black features are hyphae) Growth conditions: 6,000 *Aspergillus* spores/dish, 35°C culture, during 3 days of culture. Each data value indicates mean ± SE of duplicate independent experiments.

**Figure 4:**
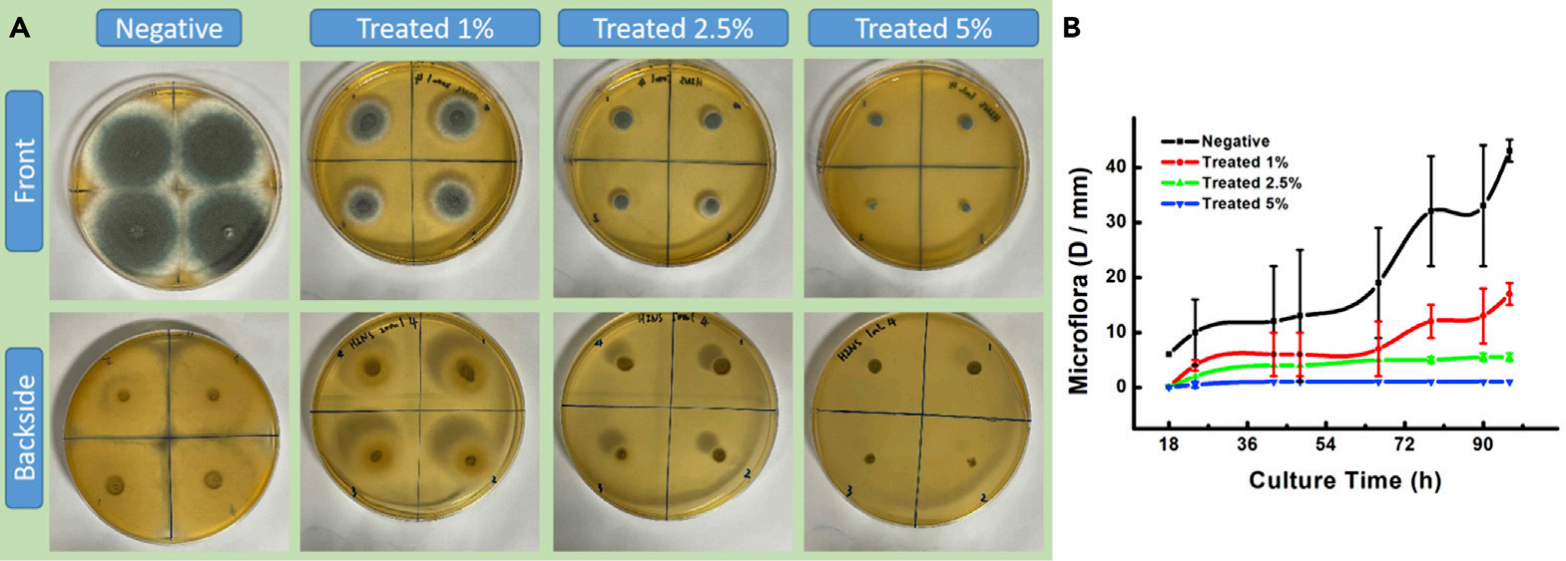
Optimization of HINS concentration for antifungal activity (A and B) (A) Photos of *Aspergillus* colonies exposed to different proportions (1.0%, 2.5%, and 5.0%) of HINS during 66 h and (B) during 90 h. Each data value indicates mean ± SE of duplicate independent experiments.

We designed an ingenious *in vitro* method to study the effect of the HINS-composed substrate. Here, we updated the reported homogeneous spread-plate culture method and the center culture method with a combination: the sterile agar was mixed with the amounts of HINS. The aspergillosis spores were planted in the center of the composite agar; and the growth condition serves as a contrast to natural *Aspergillus*, and the growth rate of the fungal spores could be calculated by measuring the surface area of the colony. Here, we traced the growth of fungal colonization (6,000 *Aspergillus* spores/dish cultured at 35°C for 3 days). We observed the proportion of the fungal colony and found that the growing tendency of the *Aspergillus* colony was almost entirely inhibited in the HINS-treated agar plate (Figure 3A). The daily collected photos recorded the hyphae growth of the fungi sensitively and clearly by the Bio-Rad ChemiDoc XPS+, as the tubular cell wall of hyphae was observed as black filiform. Owing to the principle of this image reader model is capturing the filtering light across the object; almost all the wavelengths of light could pass through the hyphae, appearing black in the image. Inversely, the saturated spores would absorb the light and appear white in the image. Furthermore, the gradient color displayed the density of spore and hyphae. By comparing the common nutrition agar (Negative) and the HINS-treated agar (Treated) in Figure 4A, we found that the hyphae of fungi could not grow on the HINS-treated agar. The colonies of the culture are summarized in Figure 3B, and the results indicated the following: (1) The hyphae were growing from the spore and then growing ahead of the spores in the common nutrition agar, which obeys the natural growth; (2) the activity of hyphae would guide the growth of *Aspergillus*; (3) in the HINS-treated agar, there was barely hyphae growth of fungi. However, the spores would survive by fission for 3 days, and the hyphae would occur as an extension at a slow speed on the surface of gathering spores. In addition, we collected the grown *Aspergillus* from those two agar media after 96 h of culture and checked their morphology by SEM. The results showed that *Aspergillus* grew strongly in the common agar plate, and numerous hyphae (Figure 1A) extended over the surface trending growth. However, in comparison, the hyphae growth of *Aspergillus* was fragile and fewer hyphae (Figure 1B) existed in the HINS-treated agar plate.

### Optimized HINS composite for the antifungal activity

Optimizing the nanomaterials is an essential property for their medical industry applications, especially the inherent toxicological issues and efficiency. To this end, we studied the three different concentrations (mixture rates: 1.0, 2.5, and 5.0%) of HINS in the composed agar (HI-AsE), and photographs recorded the growth under each condition. We monitored the fungal growth condition for 96 h. The diameter of the grown microbiota at 66 h was observed (Figure 4A). Notice that in all the culture conditions in this study, we increased the seed density to 6,000 *Aspergillus* spores/dish and set the culture room at 35°C, which would more approximate the weather condition of a tropical/subtropic rainy climate where the fungal would grow much faster. We found that the 2.5% mixture ratio decreased the growth of spores, and the 5.0% mixture ratio was sufficiently effective to inhibit the hyphae growth of fungi for 90 h and even longer, as shown by the growth trend chart (Figure 4B). The results showed that the higher the mixture ratio of HINS the greater the antifungal efficiency, which has shown persistent inhibition. Based on several studies of the mycotoxin secreted by *Aspergillus*, the hyphae growth of the fungi is caused by the apical extension tube of mycelium cells, with the release of mycotoxin.^31^^,^^32^ Therefore, blocking the hyphae growth of fungi would be desired for antifungal activity.

### Gene expression pattern for the hypersensitivity responses in HI-AsE

**Figure 5:**
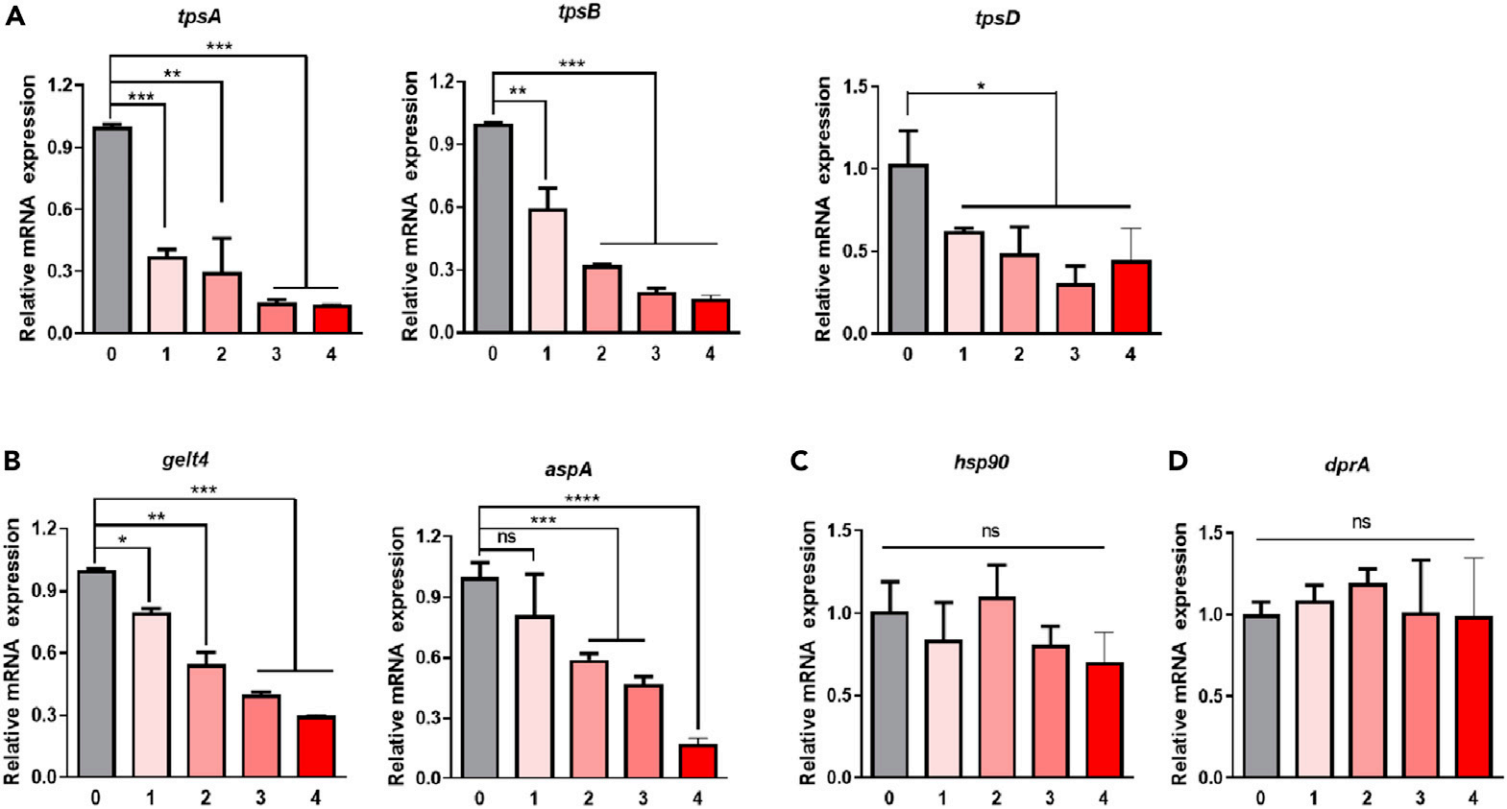
HI-AsE and Con-AsE expression patterns of genes involved in blocking the hyphae growth and survival of fungi (A) Expression of trehalose biosynthetic genes in *Aspergillus* (*tpsA*, *tpsB*, and *tpsD*). (B) mRNA expression of *gel4* (isotropic growth) and *aspA* (polarized growth) in *Aspergillus*. (C) mRNA expression of *hsp90* in response to heat stress in *Aspergillus*. (D) mRNA expression of *dprA* in response to oxidative stress in *Aspergillus*. Data are expressed as the mean ± SD. Means were compared using a one-way ANOVA, followed by Tukey’s multiple comparison tests when data were normally distributed. (0: Con-AsE, 1–4: HI-AsE concentrations 0.5, 1.0, 2.5, and 5.0%, respectively). ∗∗∗p *value (p)*< 0.001 versus negative control (NC), ∗∗∗p< 0.001 vs. negative control (NC).

To explore the mechanism by which HINS controls the hyphae growth, development, and viability of *A. fumigatus* spores, we extracted RNA from Con-AsE and HI-AsE to compare the mRNA expression levels of seven associated genes (*tpsA*, *tpsB*, *tpsD*, *gel4*, *aspA*, *hsp90*, and *dprA*). To protect itself from external stressors, such as temperature and humidity, *A. fumigatus* accumulates compatible solutes, such as trehalose.^33^ Trehalose is required for stress resistance and long-term viability. *TpsA*, *tpsB*, and *tpsD* encode enzymes involved in trehalose biosynthesis.^34^ Their expression is important for the survival of *Aspergillus* conidia.^31^^,^^34^ In the HI-AsE group, the expression levels of these genes immediately reduced compared with the Con-AsE group, indicating that HINS had a significant inhibitory effect on trehalose synthesis and impacted conidial survival (Figure 5A). Two distinct morphological changes characterize the germination of conidia: isotropic growth and polarized growth.^35^^,^^36^ Therefore, we selected *gel4* and *aspA* for isotropic growth and polarized growth of *A. fumigatus* spores, respectively.^36^^,^^37^ We found a decreasing trend of mRNA expression of *gel4* and *aspA* with the increase in HINS concentration, indicating that HINS inhibited the hyphae growth and spore development of *Aspergillus* (Figure 5B). We also tested the heat shock-related gene (*hsp90*)^38^ and oxidative stress-related gene (*dprA*)^39^ as control genes, but there was no significant difference across the groups, revealing that HINS did not alter the fungal responses to temperature changes and oxidative stress (Figures 5C and 5D). These results provide evidence that HINS inhibited the fungal hyphae growth and spore development and reduced its potential antifungal activity.

### HINS functionality in the lung hypersensitivity mouse model

**Figure 6:**
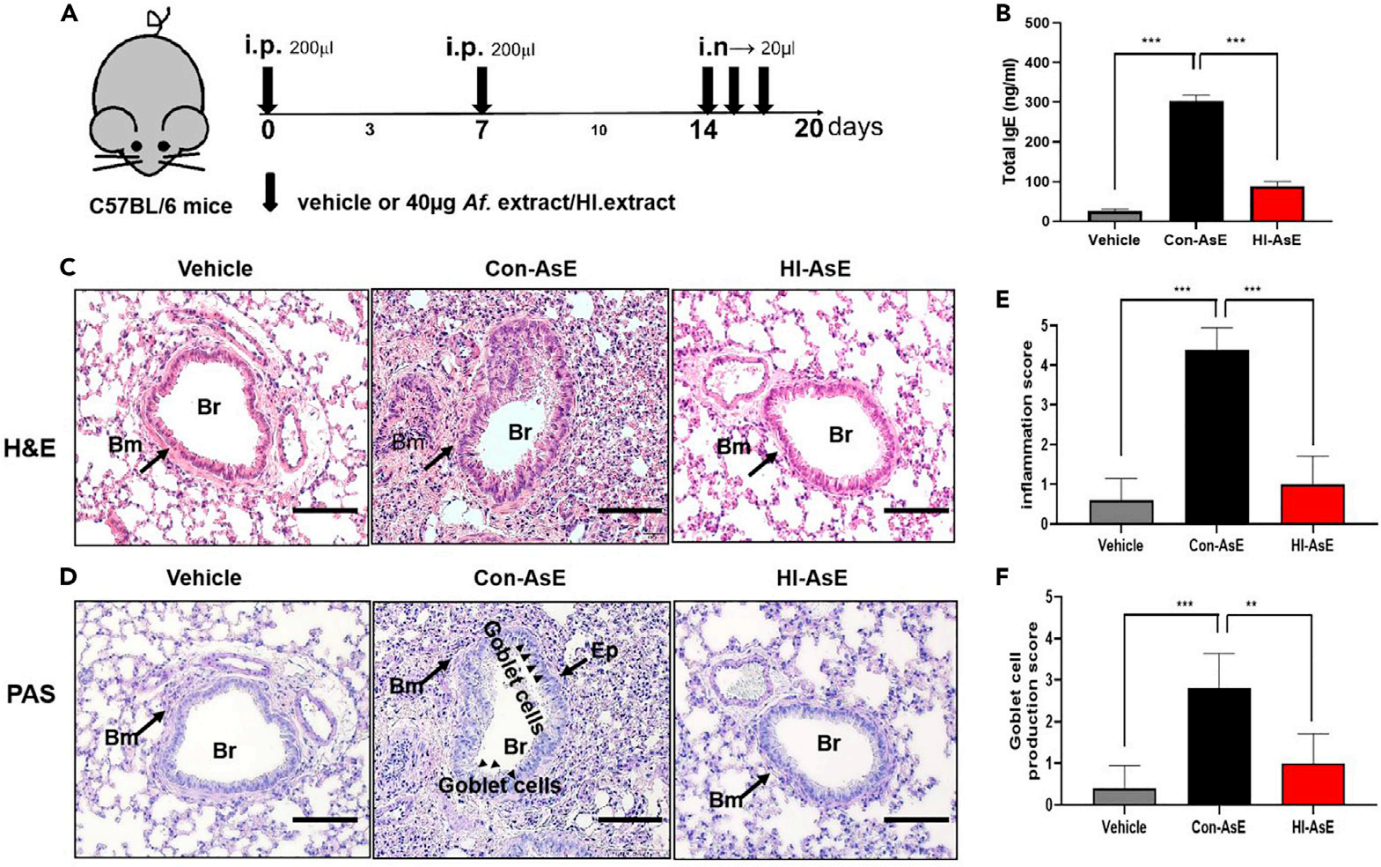
Assessment of lung histology in HI-AsE and Con-AsE mouse models (A) Experimental protocol for the development of lung disease in mice; Con-AsE or HI-AsE mouse models were induced by intraperitoneal (i.p.) sensitization and intranasal (i.n.) challenge. (B) Total IgE levels in the serum by ELISA. (C) Representative H&E-stained lung tissue sections (original magnification: ×100, zoom image of the ×10 image in the left upper quadrant, scale bar: 50 μm). (D) Representative PAS-stained sections (original magnification: ×200, scale bar: 50 μm) in control mice. (E) Assessment of the peribronchial inflammation score on histological lung slides. (F) Scoring of the goblet cell production in the three groups. Br, bronchus; Bm, basement membrane; Ep, epithelium. Scoring of the extent of mucus production in the three groups. Data are expressed as the mean ± SD Means were compared using a one-way ANOVA, followed by Tukey’s multiple comparison tests when data were normally distributed. (∗∗∗p< 0.001 versus NC, ∗∗∗p< 0.001 versus n = 5–6).

We wondered whether the theory of the HINS-treated medium preparation would have applications in medical industries. To gain insight into the attenuated pathogenesis of those fungi spores that hyphae growth from the HINS-treated agar, we evaluated the *in vivo* pathogenicity of *Aspergillus* (Con-AsE and HI-AsE) in three different mouse models. To evaluate whether HI-AsE still triggered a pathogenic response in the lung tissues of mice, we administered 40 μg of the extract products from Con-AsE or HI-AsE or vehicle to mice by i.p. 3 times for 2 weeks (Day 1, 7, and 14), followed by an i.n. challenge for three consecutive days (Day 14–16). On Day 20, mice were sacrificed for the collection of lung tissue and blood (Figure 6A). To analyze whether this induction strategy produces hypersensitivity responses in mice, the total IgE levels from mouse serum were measured. HI-AsE-treated mice had significantly lower IgE levels than those in Con-AsE mice (Figure 6B). As shown in Figure 6B, the mean total IgE level was 25.7 ng/mL in the vehicle group (PBS), 303.0 ng/mL in the Con-AsE group, and 88.5 ng/mL in the HI-AsE group, indicating that the Con-AsE treated group developed a hypersensitivity response in mice. Moreover, H&E staining for analysis of lung histopathology revealed that intratracheal administration of Con-AsE increased peribronchial and parenchymal infiltration of eosinophils in and around the airways, including significant interstitial infiltration of inflammatory cells, alveolar septal thickening, and collapsed alveolar spaces, compared with those of the vehicle and HI-AsE groups (Figure 6C). PAS staining of the lung is performed to identify goblet cell hyperplasia in the epithelium and submucosal gland hypertrophy. The Con-AsE group had significantly higher levels of airway mucus (mucin staining) compared with the vehicle and HI-AsE-treated groups (Figure 6D). The inflammatory cell (eosinophil) infiltration and goblet cell production scores were calculated (Figures 6C and 6D). Con-AsE (a ring of inflammatory cells of more than 4 cells deep) has the most inflammatory cell infiltration compared with the vehicle and HI-AsE groups (Figure 6E). The peribronchial wall inflammation was evaluated, and no score above 1 point could be noted, suggesting that no tested component in the HI-AsE group induced an inflammatory reaction in the pulmonary tissue. Goblet cell hyperplasia and airway mucus were improved in the Con-AsE group compared with the vehicle and HI-AsE groups (goblet cells were <25% in both), which reflects inflamed epithelium from Con-AsE irritation (Figure 6F). These findings indicate that we were successful in designing an AsE-induced hypersensitivity mouse model, which is consistent with other studies.^40^^,^^41^^,^^42^ Furthermore, the IgE level from mouse serum in the HI-AsE group indicated no noticeable allergic reactions, and the lung histological analysis was similar to that seen in the vehicle group. In addition, the blood biochemistry analysis (alanine transaminase, aspartate aminotransferase, bilirubin, blood urea nitrogen, and creatinine) of mice conducted 14 days after intravenous administration of HINS showed good biocompatibility. These results suggested that HINS-cultured *Aspergillus* do not have potent pathogenic functions *in vivo*, which may be due to disrupting the development of its pathogenic toxins. Therefore, HINS could be used effectively and safely in medical devices, particularly those associated with ventilators.

### HINS functionality in the skin hypersensitivity mouse model

**Figure 7:**
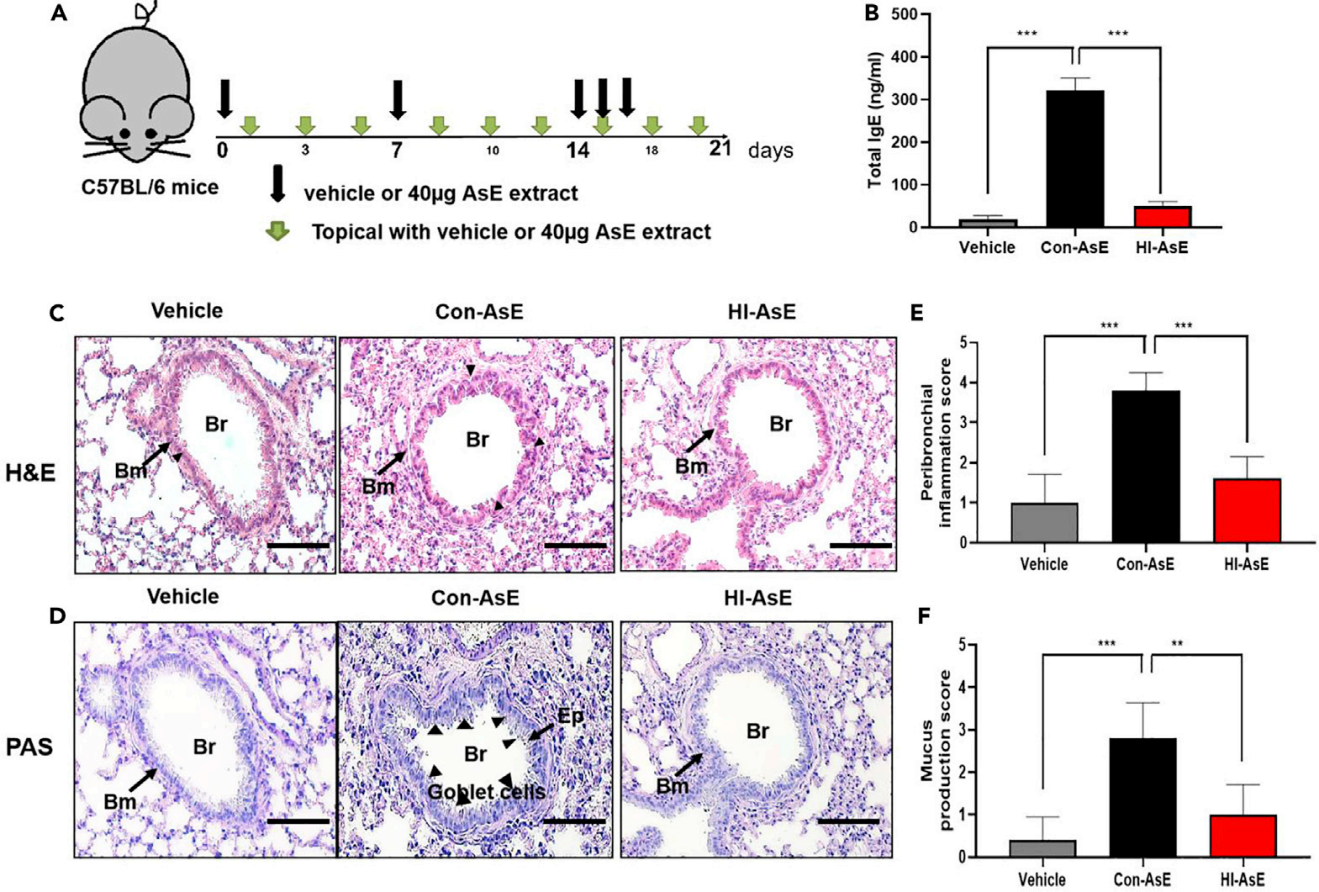
Assessment of lung histology in HI-AsE and Con-AsE mouse models (A) Experimental protocol for the development of lung hypersensitivity in mice; HI-AsE and Con-AsE mouse model induced. (B) Total IgE levels in the serum by ELISA. (C) Representative H&E-stained lung tissue sections (original magnification: ×100, magnified image of the ×10 image in the left upper quadrant, scale bar: 50 μm). (D) Assessment of the peribronchial inflammation score on histological lung slides. (E) Representative PAS-stained sections (original magnification: 200×, scale bar: 50 μm) in control mice. (F) Scoring of the extent of mucus production in the three groups. Data are expressed as the mean ± SD. Means were compared using a one-way ANOVA, followed by Tukey’s multiple comparison tests when data were normally distributed. (Original magnification: 200×, scale bar: 50 μm) (∗∗∗p< 0.001 versus NC, ∗∗∗p< 0.001 versus n = 5–6).

**Figure 8:**
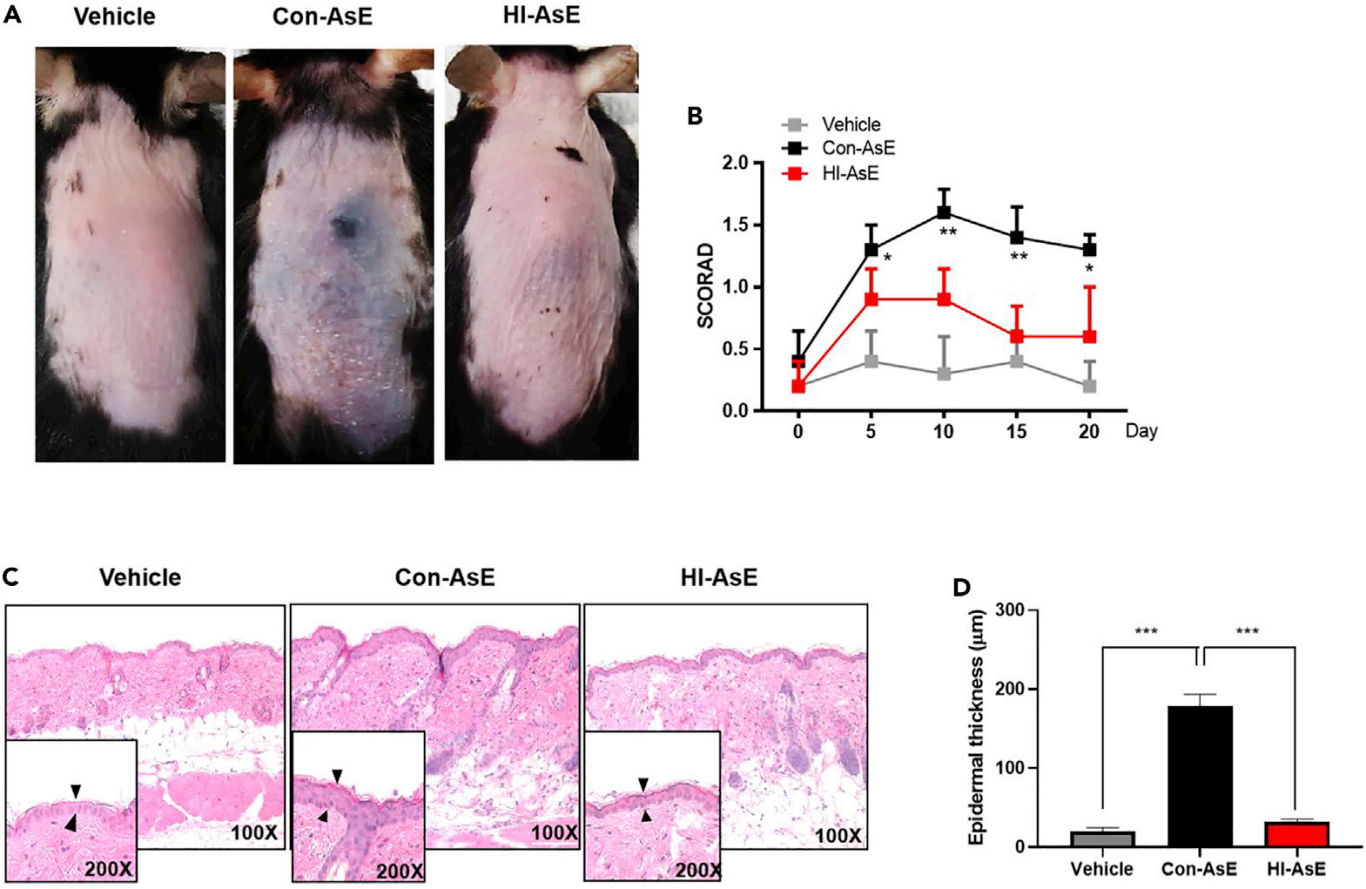
Assessment of skin histology in HI-AsE and Con-AsE mouse models (A) Macroscopic cutaneous manifestation in HI-AsE- and Con-AsE-induced groups. Skin lesions, such as erythema, erosions, crusts, and scale, were more remarkable in the HI-AsE group than in the Con-AsE group on Day 21. (B) Epidermal and dermal thickness were significantly greater in the skin from the HI-AsE-treated mice than in Con-AsE-treated mice. (C) Histopathological analysis of HI-AsE-induced AD skin. (D) Scoring of the epidermal thickness of the three groups. Data are expressed as the mean ± SD. Means were compared using a one-way ANOVA, followed by Tukey’s multiple comparison tests when data were normally distributed. (∗∗∗p< 0.001 versus NC, ∗∗∗p< 0.001 versus n = 5–6).

The term “atopic march” was created to describe the progression of atopic disorders from AD in infants to allergic rhinitis and asthma in children.^7^^,^^43^^,^^44^ To simulate this complication, the mouse epidermis was exposed to either Con-AsE or HI-AsE 3 times per week while allergic airway inflammation was induced (Figure 7A). As shown in Figure 7B, the mean total IgE level was significantly lower in the vehicle (19.1 ng/mL) and HI-AsE groups (50.1 ng/mL) than in the Con-AsE group (322.0 ng/mL). According to H&E staining, eosinophil infiltration was significantly lower in the HI-AsE group than in the Con-AsE group (Figure 7C), and lung PAS staining confirmed that the HI-AsE and control groups had similar levels of airway mucus (Figure 7D). Based on the quantification of histological staining and scoring criteria, inflammatory cell infiltration in the Con-AsE group (nearly 4 points) was more than that in the vehicle (1 point) and HI-AsE groups (<2 points). Goblet cells produced by the HI-AsE group (<1 point) were almost comparable to the vehicle group (Figures 7E and 7F). The results are consistent with the theoretical report of the pathogenesis-related protein located on hyphae of fungi.^7^^,^^29^ In addition, the Con-AsE group developed significant epidermal dryness and AD symptoms after 20 days, whereas this inflammatory symptom was not observed in the skin of the HI-AsE- or vehicle-treated groups (Figure 8A). Based on the SCORAD index, the skin of the Con-AsE group had the most noticeable inflammatory score on Day 10, whereas the HI-AsE group had no significant changes in the inflammatory signature over the 3-week treatment period (Figure 8B). On Day 21, histological studies of the lesional skin from mice revealed significant hyperplasia of the epidermis in the Con-AsE group compared with the HI-AsE- and vehicle-treated groups (Figure 8C). Epidermal thickness of the Con-AsE group was also significantly greater than that of the HI-AsE- and vehicle-treated groups (Figure 8D). These findings demonstrate that HI-AsE does not sensitize or trigger pathogenic reactions in the skin or lungs, thus enhancing the applicability of HINS materials in daily life for both breathing apparatus and skin-contact-related home amenities. Collectively, our data confirmed that the use of HINS composites within the safe concentration range could not only inhibit the growth of hyphae and reduce the pathogenic source but also decrease the use of antibiotics for prophylaxis or treatment of invasive aspergillosis.

### Conclusion

In our study, we stimulated the skin and lung tissues with the AsE antigen for the first time, simulating the *Aspergillus*-induced skin and lung hypersensitivity animal model. Currently, many research groups are working on optimizing the performance of ZnO by the surface modification methods. Among these, studies on biomolecular modification with the benefits of biocompatibility and nontoxicity are promising in therapy applications. This work has provided a strong framework for future studies on the mechanism of *Aspergillus* sensitization. First, pneumonia and pneumonia−skin disease complication models were induced successfully with the safe and non-active AsE. Compared with the traditional dust induction method, it is easier to control the state of lesions to achieve guiding significance. Second, our *in vitro* experiments observed that the HINS complex successfully inhibits the fungi growing at a 5.0% mixture ratio for the HINS-treated agar, and with the elongation of the treatment time, the effect of arresting the mycelium was significantly noticeable. Drug resistance development and pathogenicity are associated with biofilm formation by fungal pathogens. In this context, we believe that as a prophylactic measure against pathogenic fungi, promoting the HINS composite substance for packaging of daily stuff, construction materials, and medical instruments (Scheme 1) could provide clean auxiliary medical equipment for patients undergoing disease treatment. Furthermore, we discovered that HI-AsE does not trigger any significant allergic response in mouse lung and skin tissues by analyzing and comparing extracts collected from Con-AsE-treated and HI-AsE-treated mice. This may be because of the inhibition of the development of toxicity in the hyphae of *Aspergillus* by HINS composites. Through gene expression analysis (*tpsA*, *tpsB*, *tpsD*, *gel4*, *aspA*, *hsp90*, and *dprA*) in the Hi-AsE and Con-AsE groups, we showed the HINS composite inhibited growth of hyphae, which would attenuate the hypersensitivity responses. Moreover, the HINS composites show high biological compatibility with few side effects when used in a safe concentration and thus could be used in medical materials and devices to provide clean auxiliary medical equipment for patients undergoing disease treatment.

The mycelium tip shows a gradient growth pattern, and the mycelium tip expands the fastest. Hyphal swelling and numerous hyphae were key features of *Aspergillus* cultured in common nutrition agar and may be indicative of mycotoxin biosynthesis and pathogenicity. Using the HINS composite within the safe concentration range could not only inhibit the growth of hyphae but also reduce the number of fungal pathogens. However, the industry-scale production and stability of HINS are understudied, and the durability of its effectiveness is an interesting topic for future study. We believed that the HINS would be used in producing safety equipment, kits, with prophylactic and therapeutic agents against pathogenic fungi. We envision the HINS composites will be used in antibiotic applications combined with the research on the physical and chemical properties of theriacs providing the challenges of studying the functional mechanism of HINS and other nanomaterials for further applications, including the protection of genetic diversity and medical safety, are addressed.

### Limitations of the study

Like every coin has two sides and the effect of microbes on humans will always be an ambiguous topic without a precise definition. On the one hand, human immune responses to fungi are intricate, starting from innate (non-specific) immune reactions to particular acquired immune responses induced during pathogen infestations, which have encouraged the study of human antifungal immune reactions and the respect for biodiversity. On the other hand, controlling fungi infestations requires understanding the growth and pathogenic mechanisms. Our model remains unable to fully reflect actual fungal skin exposure in a normal human environment in terms of viability, amount, and life cycle of *Aspergillus*; optimization of this experimental approach in a future study would facilitate further research on pathogenic mechanisms and provide constructive experimental evidence for the development of therapeutic drugs. Meanwhile, the study on genetic and histological analysis of infectious mouse model is quite novel which encourages us to pay more attention to studying their specificity and immune mechanisms.

## STAR★Methods

### Key resources table

**Table:** 

REAGENT or RESOURCE	SOURCE	IDENTIFIER
**Bacterial and virus strains**		

*Aspergillus fumigatus*	ATCC	ATCC 36607

**Chemicals, peptides, and recombinant proteins**		

Zinc nitrate hexahydrate (ZnNO_3_•6H_2_O, 98%)	Sigma	N/A
Ammonium hydroxide solution (28%)	Sigma	N/A
Dimethyl pimelimidate dihydrochloride	Sigma	D8388-5G
Dimethyl suberimidate dihydrochloride	Sigma	179523-5G
Hematoxylin and eosin stain	Sigma	CAS No.: 517-28-2 and 56360-46-4
Sodium dodecyl sulfate	Sigma	CAS No.: 151-21-3
Cetyltrimethylammonium bromide	Sigma	CAS No.: 57-09-0

**Critical commercial assays**		

Fungal Sample Preparation Kit	InfusionTech, Korea	N/A
AccuPower RT PreMix Kit	Bioneer, Korea	N/A
EcoDry Premix Kit	Takara, Japan	N/A
ELISA Kit	San Diego, CA, USA	Cat #432404

**Deposited data**		

Raw and analyzed data	This paper	iScience

**Experimental models: Organisms/strains**		

C57BL/6 mice	Bar Harbor, ME, USA	Eight-week-old

**Oligonucleotides**		

tpsA Gene F: CAATCCTTGGAATACGGAAGAG R: GTCCAGTTTGGAGAAGTTGAGG	MACROGEN	N/A
tpsB Gene F: GGGTGGTCTGGTAACTGGTTT R: ACCGATCCAAGTTCATCCTCT	MACROGEN	N/A
tpsD Gene F: TACGTTGCAGAAGCAAGAGGT R: AGGCCCTTGATGTAGTCCAGT	MACROGEN	N/A
dprA F: TACATATGACAGAGGACGAACGTTTCG R: ATCTCGAGTCAAGTTCGGATACGCTGGT	MACROGEN	N/A
hsp90 F: CGTCAAGTCCATCACTCAGC R: GCTTGTGGATGCGCTCGGC	MACROGEN	N/A
gel4 F: AACTCCGACTACACCGATCC R: AGGTACGGATGACATTGGTG	MACROGEN	N/A
aspA F: ATGTCGTCCGCCTACAACCCG R: CTGCTCGGCCTCGGCGGCTTC	MACROGEN	N/A
Actin F: TGCCCTTGCTCCCTCGTCTA R: ACCGCTCTCGTCGTACTCCT	MACROGEN	N/A

**Software and algorithms**		

GraphPad Prism 8	San Diego, CA, USA	Version 8.2
Meta-Morph image	Sunnyvale, CA, USA	Molecular Devices
ImageJ	Figures 3 and 4	https://imagej.nih.gov/ij/
BioRender	Figure 1A	https://biorender.com/

### Resource availability

#### Lead contact

Further information and requests for resources and reagents should be directed to and will be fulfilled by the lead contact, Yong Shin (shinyongno1@yonsei.ac.kr).

#### Materials availability

Materials are available up on request.

### Method details

#### Chemicals and reagents

All experimental reagents were analytically pure and used without further purification. Zinc nitrate hexahydrate (ZnNO_3_•6H_2_O, 98%), ammonium hydroxide solution (28% NH_3_ in H_2_O, 99.99% trace metal reference), dimethyl pimelimidate dihydrochloride (DMP, Sigma, D8388-5G), dimethyl suberimidate dihydrochloride (DMS, Sigma, 179523-5G), hematoxylin and eosin stain (H&E, Sigma, CAS No.:517-28-2 and CAS No.:56360-46-4), and periodic acid−Schiff (PAS, Sigma, CAS No.:10450-60-9) were purchased from Sigma-Aldrich (St. Louis, MO, USA). Sodium dodecyl sulfate (SDS, Sigma, CAS No.: 151-21-3) was bought from Welgene (Daegu, Korea). Cetyltrimethylammonium bromide (C_19_H_42_BrN, >98%, CTAB, Sigma, CAS No.: 57-09-0) was purchased from Tokyo Chemical Industry Co., Ltd. (Tokyo, Japan). Sabot’s dextrose agar and chloramphenicol medium containing chloramphenicol medium (Cat #C6781; Lot #437412) were purchased from Santa Maria. Milli-Q water and phosphate-buffered saline (PBS, 10X, pH 7.4, Thermo Fisher Scientific, CAS No.: 12579099) were used in all the experiments.

#### Biological samples

*A. fumigatus* ATCC 36607 was grown in Sabouraud dextrose agar at 25°C for 5 days. After culture, the suspension was centrifuged at 1,400 g for 10 minutes. The pellet of the *Aspergillus* fungus was re-suspended in PBS and quantified using a hemocytometer. The HINS treatment *Aspergillus* were collected using three different methods (−56°C dry ice and dried at room temperature [RT] for live *Aspergillus*, autoclaved at 121°C for 15 min for non-active *Aspergillus*). To test the effect of HINS nanomaterials on fungi, we prepared the glucose agar medium plates and the specific solid medium. Fungal spores after 5-day culture were collected (600 spore/20 μL) in PBS solution to obtain uniformity and reproducibility in the experiment. The 20 μL of spore seed solution has been dropped uniformly on the center of the agar medium plates with the different concentrations of HINS, dried, and placed upside down. The plate was kept at 36°C for 3 days, and a photographic record of each fungal sample was taken. Image-J was used to analyze the records to compare the growth rate of each HINS samples with the control sample.

#### Preparation of the HINS composite

To enhance the stability and biocompatibility of ZnO-NS, a homobifunctional imidoester (HI, DMS) modification was carried out according to the previous study.^26^ Briefly, 4 mg ZnO-NS (40 mg/mL, 100 μL) and 4 mg HI (DMS, 10 mg/mL, 400 μL) were dissolved in a 1.5 mL EP tube in a 500 μL solution. The mixture was oscillated by an oscillating machine (Magic-mixer TMM-5). After 20 min, the mixture was centrifuged at 12,000 rpm for 5 min in a mini-centrifuge (LaboGene). The supernatant liquid was removed, and the precipitate was washed twice with Milli-Q water. Finally, 400 μL of Milli-Q water was used to re-suspend the precipitate to obtain 10 mg/mL of HINS composite solution. The precipitate was dried overnight in a drying oven (DX312C, Yamato) at 56°C.

The ZnO-NS crystals were synthesized by the hydrothermal method in an alkaline medium, as proposed in our previous work. Accordingly, 1 mL of 1 M Zn(NO_3_)_2_•6H_2_O (Sigma, 228737-100G) and 1 mL of 1 M CTAB were added to 98 mL of Milli-Q water in a 250 mL flask. The mixture was magnetically stirred (500 rpm) while heating at 95°C for 50 min. Subsequently, under steady stirring conditions, 2 mL of ammonium hydroxide solution was added dropwise to the reaction mixture. After stirring, a milky colloidal solution appeared. To stop the growth of NS-ZnO, the reaction flask was immediately placed in a 0°C freezer. After nearly 10 min, the product was transferred to a 50 mL tube, and the solution was centrifuged. The supernatant liquid was removed, and the precipitate was re-suspended in 50 mL Milli-Q water to wash out the residual ions. This process was repeated 3 times. Finally, the precipitate was dried overnight in a drying oven (DX312C, Yamato) at 56°C.^26^

#### Characterization of composite

A field emission-scanning electron microscope (FE-SEM, JSM-7500F JEOL), high-resolution transmission electron microscope (TEM, SU900; operating at a band gap of 0.8 eV and acceleration voltage of 300 kV), and optical microscope (Eclipse Ts2-FL, Nikon) were used to determine the surface morphology and characteristics of the HINS samples. The dynamic light scattering technique was used to measure the zeta potential of the material and its modified equivalent on the DynaPro NanoStar instrument (Wyatt). Light microscopy was performed by the Leica Microsystems (Ltd., Milton Keynes, UK; magnification, ×100 and ×200). Then, the basic characterization studies (UV-Visible, XRD, FTIR, and EDX) of HINS composite has been performed in the previous study.^26^

#### Real-time quantitative PCR (qPCR)

Total RNA was extracted using the Fungal Sample Preparation Kit (InfusionTech, Anyang, Korea) in accordance with the manufacturer’s recommendations. The quality and quantity of RNA were analyzed by determining the ratio of ultraviolet (UV) absorbance at 260 nm–280 nm. cDNA was synthesized from total RNA using the AccuPower RT PreMix Kit (Bioneer, Daejeon, Korea). To this end, 0.1 ng−1 μg of template RNA and 12 μL of nuclease-free water were added to each tube containing dNTPs buffer to synthesize cDNA using EcoDry Premix Kit (Takara, Kusatus, Shiga Prefecture, Japan). The samples were combined, briefly centrifuged, then incubated at 42°C for 60 min, followed by 70°C for 10 min. The synthetic cDNA product was used for qPCR. The expression levels of *tpsA*, *tpsB*, *tpsD*, *dprA*, *hsp90*, *gel4*, and *aspA* genes were assayed using specific primers designed using Primer Blast and synthesized by Bioneer (Table 1). qPCR was performed using Power SYBR® Green PCR Master Mix (Thermo Fisher Scientific, Waltham, MA, USA) on an Applied Biosystems 7500 StepOne Plus Real-Time PCR System. PCR conditions were as follows: initial denaturation at 95°C for 30 s; followed by 40 cycles of denaturation (95°C for 10 s), annealing (58°C for 30 s), and extension (72°C for 20 s). *Actin* was used as an internal reference gene, as described previously.^35^ The results were analyzed using GraphPad Prism 8 (version 8.2; GraphPad Software, Inc., San Diego, CA, USA), and the gene expression levels were calculated as relative fold changes using the 2^−ΔΔ*Ct*^ method. Each experiment was performed in triplicate.

**Table 1:** Primer list of *Aspergillus* (RNA) amplification

Primer list	
Gene	Primer sequence (5′ to 3′)
*tpsA*	F: CAATCCTTGGAATACGGAAGAG
	R: GTCCAGTTTGGAGAAGTTGAGG
*tpsB*	F: GGGTGGTCTGGTAACTGGTTT
	R: ACCGATCCAAGTTCATCCTCT
*tpsD*	F: TACGTTGCAGAAGCAAGAGGT
	R: AGGCCCTTGATGTAGTCCAGT
*dprA*	F: TACATATGACAGAGGACGAACGTTTCG
	R: ATCTCGAGTCAAGTTCGGATACGCTGGT
*hsp90*	F: CGTCAAGTCCATCACTCAGC
	R: GCTTGTGGATGCGCTCGGC
*gel4*	F: AACTCCGACTACACCGATCC
	R: AGGTACGGATGACATTGGTG
*aspA*	F: ATGTCGTCCGCCTACAACCCG
	R: CTGCTCGGCCTCGGCGGCTTC
*actin*	F: TGCCCTTGCTCCCTCGTCTA
	R: ACCGCTCTCGTCGTACTCCT

#### *In vivo* testing on sensitization

Eight-week-old female C57BL/6 mice, weighing approximately 22–28 g, were purchased from Jackson Laboratory (Bar Harbor, ME, USA). The mice were housed for routine breeding in specific pathogen-free facilities at the Yonsei University College of Medicine with a 12-h light and 12-h dark cycle environment and *ad libitum* access to food and water. All animal experiments were performed in accordance with the ethical guidelines for animal studies and were approved by the Institutional Animal Care and Use Committee of Yonsei University College of Medicine (IACUC No. 2020-0035). The mice were allowed to acclimatize for 1–2 weeks before starting the experimental protocol and were monitored daily for general health. For mice lung infection with *Aspergillus*, because the *Aspergillus*-induced allergy mouse model is currently in the exploratory stage, we performed experiments based on the approaches described.^21^^,^^45^

Mice were treated intraperitoneally (i.p.) with Con-AsE and HI-AsE at 40 μg/200 μL in PBS on Day 0, 7, and 14 to initiate the immune response. Mice were then challenged i.n. with Con-AsE and HI-AsE in the same amount for three consecutive days on Day 14, 15, and 16. Negative controls were mice not exposed to *Aspergillus* extract (AsE) but treated with PBS only as a vehicle group. After mice were euthanized on Day 21, whole blood, skin, and lung tissues were extracted for analysis. For the AD-like skin inflammation mouse model, mice were acclimatized for 1-week. Then, the upper back hair was removed from anesthetized mice using an electric shaver and a depilation procedure. The skin barrier was disrupted using 200 μL of 4% SDS. After 2 h, the shaved dorsal skin surface was treated with 40 μg of dried non-active Con-AsE or HI-AsE. The negative control group was coated with PBS only as a vehicle. Inductions were performed 3 times per week for three consecutive weeks.

#### Evaluation of dermatitis severity

The scoring atopic dermatitis (SCORAD) index was used in mice to assess the severity of dermatitis symptoms.^46^ This index is determined by the presence of erythema/bleeding, scarring/dryness, edema, and flaking/erosion. Each symptom is assigned a score of 0 (none), 1 (mild), 2 (moderate), or 3 (severe). The total SCORAD score is the sum of each individual’s scores.^47^ Two independent researchers evaluated each score on a weekly basis. Pictures were captured with the same camera settings and lighting every week.

#### Histological analysis for lung tissue and skin tissue

For histological examination, lung tissue was removed and fixed in a 10% (v/v) neutral-buffered formalin solution. Lung tissue was embedded in paraffin. Sections were cut at 4 μm thickness and stained with H&E and PAS.^5^^,^^48^ The extent of inflammatory cell influx, mucus production, and goblet cell invasion was observed under a light microscope (Magnification, ×100 and ×200). Peribronchial infiltration and goblet cell hyperplasia were assessed by semi-quantitative scores (0–5), as reported previously.^49^ To grade peribronchial inflammatory cell infiltration, the peribronchial cell count was determined based on the following 5-point grading system: 0, normal; 1, few cells; 2, a ring of inflammatory cells one cell layer deep; 3, a ring of inflammatory cells of 2–4 cells deep; 4, a ring of inflammatory cells of more than 4 cells deep.^50^^,^^51^ The extent of mucus production was assessed by PAS staining, and goblet cell hyperplasia in the airway was scored as follows: 0, no goblet cells; 1, <25%; 2, 25–50%; 3, 50–75% (including 50%); 4, >75%. Five-to-six fields of view were counted per stained tissue sample, and the mean score was calculated for 5 mice per group. Measurements were scored by 2 experimenters in a blinded manner. The dorsal skin tissue of each group of mice was cut with a sterile scalpel and fixed with 4% paraformaldehyde for H&E staining. Epidermal thickness was measured by Meta-Morph image analysis software (Molecular Devices, Sunnyvale, CA, USA), and eosinophils were counted using an Olympus BX53 microscope (Olympus Corp., Tokyo, Japan). Three different images were taken from each slide.^46^

#### Measurement of sera immunoglobulins by enzyme-linked immunosorbent assay (ELISA)

Whole blood was collected from mice before sacrifice, and serum samples were collected by centrifugation at 4,000 rpm, 4°C for 15 min. Total immunoglobulin E (IgE) levels in mouse serum were measured from an initial serum dilution of 1/10 in PBS+1% bovine serum albumin using an ELISA kit (BioLegend, San Diego, CA, USA, Cat #432404) according to the manufacturer’s instructions.
